# Large-scale DNA methylation expression analysis across 12 solid cancers reveals hypermethylation in the calcium-signaling pathway

**DOI:** 10.18632/oncotarget.14417

**Published:** 2017-01-02

**Authors:** Xiao-Xiong Wang, Fu-Hui Xiao, Qi-Gang Li, Jia Liu, Yong-Han He, Qing-Peng Kong

**Affiliations:** ^1^ State Key Laboratory of Genetic Resources and Evolution, Kunming Institute of Zoology, Chinese Academy of Sciences, Kunming 650223, China; ^2^ Kunming College of Life Science, University of Chinese Academy of Sciences, Kunming 650204, China

**Keywords:** DNA methylation, gene expression, pan-cancer, calcium-signaling pathway

## Abstract

Tumorigenesis is linked to the role of DNA methylation in gene expression regulation. Yet, cancer is a highly heterogeneous disease in which the global pattern of DNA methylation and gene expression, especially across diverse cancers, is not well understood. We investigated DNA methylation status and its association with gene expressions across 12 solid cancer types obtained from The Cancer Genome Atlas. Results showed that global hypermethylation was an important characteristic across all 12 cancer types. Moreover, there were more epigenetically silenced than epigenetically activated genes across the cancers. Further analysis identified epigenetically silenced genes shared in the calcium-signaling pathway across the different cancer types. Reversing the aberrant DNA methylation of genes involved in the calcium-signaling pathway could be an effective strategy for suppressing cancers and developing anti-cancer drugs.

## INTRODUCTION

Cancer is a complex disease [1, 2], with the dysregulation of genes linked to many tumor features [3, 4]. Of these cancer-associated genes, oncogenes are usually activated, whereas tumor suppressor genes are inactivated [5]. A diverse number of regulators control gene expression in cancer, including genetic and epigenetic changes [6–8]. In recent years, epigenetics in cancer research has attracted considerable attention due to its emerging role in cancer development, prognosis, and treatment [5, 9, 10]. As the most common epigenetic modification, and as a bridge between gene expression regulation and chromatin architecture [11, 12], DNA methylation is crucial for gene and transposon silencing and imprinting and X chromosome inactivation [13]. DNA hypermethylation in gene promoter regions leads to gene downregulation, whereas hypomethylation causes gene upregulation [11, 14]. Alteration of the site-specific targeted regions of gene promoters can upregulate gene expression in cancer cells [15]. Abnormal DNA methylation often occurs in cancers, which regulates the expression of genes, especially those responsible for cell growth, proliferation, differentiation, and apoptosis processes [10, 11, 16].

Indeed, aberrant DNA methylation is a common feature of cancer cells and is correlated with gene expression [17–21]. A recent study reclassified cancers into different methylation-driven subgroups, providing new insights into cancer and strategies for cancer diagnosis, prognosis, and therapy [14, 22–24]. Given its heterogeneous nature, however, much is still unknown about cancer, including whether a common pattern of DNA methylation exists across various cancer types.

In this study, we analyzed the associations of pan-cancer DNA methylation and gene expression in 4,138 cancer tissue samples and 338 matched normal tissue samples across 12 solid cancer types. We found that the number of lowly expressed genes with hypermethylation (epigenetically silenced genes) ranged from 33 to 992, and the number of highly expressed genes with hypomethylation (epigenetically activated genes) ranged from 0 to 34. Interestingly, the epigenetically silenced genes were found to be enriched in the calcium-signaling pathway across nine cancer types. These results suggest that various cancers share similarities in DNA methylation, with more epigenetically silenced than epigenetically activated genes identified across cancers, and reversing aberrant DNA methylation of genes involved in the calcium-signaling pathway could be a potential strategy for cancer treatment.

## RESULTS

### Global hypermethylation across 12 cancer types

**Figure 1:**
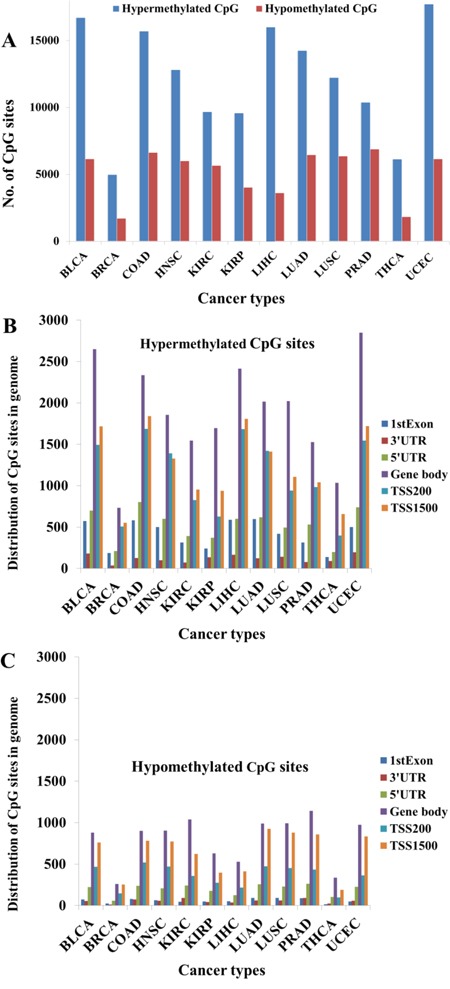
Methylation status across 12 cancer types **A**. Global hypermethylation across 12 cancer types. **B**. Distribution of hypermethylated CpG sites in the genome across 12 cancer types. **C**. Distribution of hypomethylated CpG sites in the genome across 12 cancer types.

We analyzed a total of 485,577 CpG sites in each tissue sample across 12 solid cancer types. As shown in Figure 1, the number of hypermethylated and hypomethylated CpG sites ranged from 4,972 to 17,724 and 1,705 to 6,870, respectively, among the cancer types. Thus, the number of hypermethylated CpG sites was around 2.5 times greater than that of the hypomethylated CpG sites (Figure 1A). Approximately half of the CpG sites could be annotated and were distributed in different regions in each of the 12 cancers, including the 1st Exon, 3’UTR, 5’UTR, gene body, TSS200, and TSS1500 regions, with the highest distribution in the gene body and TSS1500 regions, followed by the TSS200 region, and lastly the 3’UTR region (Figure 1B, 1C). Furthermore, for most CpG sites, there were twice as many hypermethylated CpG sites annotated in the genome regions than there were hypomethylated sites (Supplementary Table 2).

### Epigenetically silenced and activated genes across the 12 cancer types

**Figure 2:**
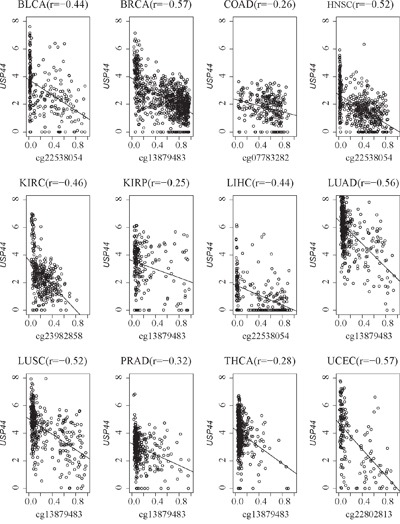
Negative correlation of *USP44* gene expression and DNA methylation across 12 cancer types X-axis from 0 to 1 represents the beta value of the *USP44* CpG sites in each cancer type. Y-axis from 0 to 8 represents the *USP44* gene expression value (log_2_(RSEM+1)) across 12 cancer types.

Many of the CpG sites were negatively correlated with gene expression (r < -0.2, p < 0.05; Supplementary Table 3). For example, *USP44* gene expression was significantly negatively correlated with DNA methylation across all cancer types (all r < -0.2, p < 0.05; Figure 2). According to the correlation coefficients, we counted the number of hyper- or hypomethylated genes in each cancer type. As shown in Table 1, the number of downregulated genes ranged from 33 to 992 among the cancer types. Of these genes, 6 were epigenetically silenced in nine cancer types, 37 were epigenetically silenced in at least eight cancer types, 96 were epigenetically silenced in at least seven cancer types, 190 were epigenetically silenced in at least six cancer types, and 340 were epigenetically silenced in at least five cancer types (Supplementary Table 4). For instance, *NOVA1*, *NRXN1*, *TMEM132C*, *USP44*, *VIPR2*, and *ZSCAN23* were consistently downregulated in nine cancers (Supplementary Table 5). It is possible, therefore, that some of these silenced genes could be good biomarkers for cancer prognosis. For example, the highly expressed *ZSCAN23* gene was associated with good prognosis in BRCA (p = 0.004, Supplementary Figure 1). The number of activated genes with hypomethylation (epigenetically activated genes) ranged from 0 to 34 across cancers (Table 1). There were only four upregulated genes with low DNA hypomethylation across two cancer types. *GRHL2* was activated in BRCA and UCEC; and *NAA25*, *NOD2*, and *TNFRSF9* were activated in KIRC and KIRP (Supplementary Table 6).

**Table 1:** Number of genes regulated by DNA methylation in 12 cancer types

Cancer types	Epigenetically silenced genes	Epigenetically activated genes
COAD	992	0
UCEC	974	21
LIHC	818	0
BRCA	677	4
LUAD	673	0
LUSC	659	4
BLCA	581	0
HNSC	579	2
KIRC	414	29
KIRP	317	34
PRAD	299	3
THCA	33	3

### Hypermethylation in the calcium-signaling pathway across different cancer types

**Figure 3:**
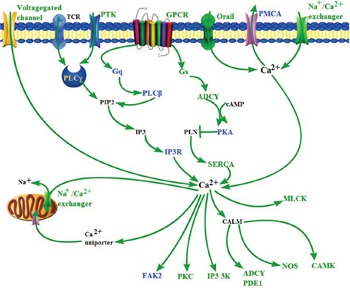
Epigenetically silenced genes of the calcium-signaling pathway in nine solid cancer types (BLCA, BRCA, COAD, HNSC, KIRP, LIHC, LUAD, LUSC and UCEC) Epigenetically silenced genes of the calcium-signaling pathway were based on the KEGG pathway database. Green proteins were downregulated by hypermethylation in more than two solid cancer types. Light blue proteins were downregulated by hypermethylation in one cancer type. Black proteins were not downregulated by hypermethylation in any cancer type.

**Figure 4:**
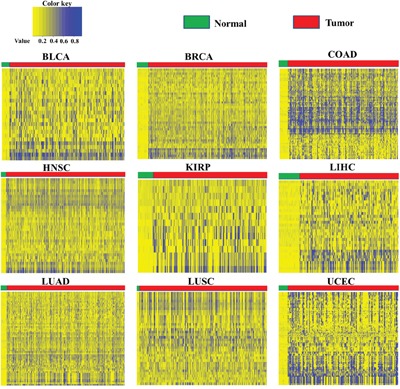
DNA methylation of epigenetically silenced genes involved in the calcium-signaling pathway across nine cancer types Green lines of each small figure represent normal tissue and red lines represent tumor tissue of each cancer type. Upper column represents the DNA methylation level from low to high.

We analyzed enrichment of epigenetically silenced genes in each cancer type. The calcium-signaling pathway was shared in nine cancer types, followed by the cell adhesion pathway shared in eight cancer types, and the cancer and Wnt signaling pathways shared in five cancer types (Table 2). To validate these results, based on the 340 epigenetically silenced genes in at least five cancer types (Supplementary Table 4), we drew a protein-protein interaction network and found some genes with higher degrees of interaction, such as *ADCY4*, *ADCY8*, and *PRKCB*. (Supplementary Figure 2). In addition, enrichment analysis of biological pathways showed that the 340 epigenetically silenced genes were enriched in the neuroactive ligand-receptor interaction, calcium-signaling, cell adhesion molecules (CAMs), vascular smooth muscle contraction, and melanogenesis pathways (Supplementary Table 7). Of note, many genes involved in the calcium-signaling pathway were epigenetically silenced. For example, *AGTR1*, *GRIN2A*, *ITPKB*, and *SLC8A3* were repressed by hypermethylation in six cancer types, and *ADCY4*, *ADCY8*, *BST1*, and *PRKCB* were inhibited by hypermethylation in five cancer types (Supplementary Table 8). Furthermore, GPCR, membrane Na^+^/Ca^2+^ exchanger, MLCK, and CAMK were decreased by hypermethylation in at least three cancer types, and most other genes (voltage-gated channel, PTK, Gq, PLC_β_, Gs, PMCA, IP3R, PKA, SERCA, FAK2, PKC, IP3K and NOS) were downregulated in at least one cancer type (Figure 3). The genes downregulated by hypermethylation were located in nodes of the calcium-signaling pathway, such as Na^+^/Ca^2+^ exchanger and GPCR (Figure 3). Moreover, the DNA methylation of genes in the calcium-signaling pathway clearly distinguished the cancer samples from the normal tissue samples across nine cancer types (Figure 4), providing further support for DNA methylation in gene expression regulation in the calcium-signaling pathway in cancer cells. We also analyzed gene expression fold change of eight genes in the calcium-signaling pathway across twelve cancer types (Supplementary Table 9), and found that these genes were almost all downregulated across cancers.

**Table 2:** Pathway enrichment of epigenetically silenced genes across different cancer types

Pathways	Cancer types
hsa04020: Calcium signaling pathway	BLCA, BRCA, COAD, HNSC, KIRP, LIHC, LUAD, LUSC, UCEC
hsa04514: Cell adhesion molecules (CAMs) pathway	BLCA, BRCA, COAD, HNSC, LIHC, LUAD, PRAD, UCEC
hsa05200: Pathways in cancer	BLCA, COAD, LIHC, LUSC, PRAD
hsa04310: Wnt signaling pathway	BLCA, COAD, KIRC, LIHC, LUAD
hsa04062: Chemokine signaling pathway	COAD, LIHC, LUAD
hsa04510: Focal adhesion pathway	BLCA, LIHC, UCEC

## DISCUSSION

Abnormal gene expression and aberrant DNA methylation are common characteristics in cancer cells [1, 2]. In this study, we investigated DNA methylation patterns and their potential role in regulating gene expression across 12 solid cancer types. Results showed that more epigenetically silenced than epigenetically activated genes were detected across cancers. This might be caused by the different number of hypermethylated and hypomethylated CpG sites (Figure 1A). In addition, more hypermethylated CpG sites locate closely to the gene promoter regions (Figure 1B, 1C). This distribution of CpG sites is consistent with previous findings that cancer exhibits hypomethylation in many repeat sequences in the genome, but displays hypermethylation in local regions, especially in gene promoter regions [25, 26]. Hypermethylated CpG sites near the gene promoter regions can lead to gene silencing in cancer [26–28], so differences in the number of hypermethylated or hypomethylated genes would be expected. Generally, the level of global hypomethylation in the whole cancer genome is mainly due to low methylated CpG sites located in the intergenic regions, including the repeated DNA sequences [25]. In addition, hypomethylation in the gene body region might regulate gene splicing [20]. Furthermore, methylation disorders have been linked to low-level gene expression in cancer cells [29]. In fact, highly expressed genes in cancers are preferentially regulated by other epigenetic factors, such as microRNA, in diverse cancer types [9, 19, 30].

Compared with previous research [22, 31], we found that the commonly dysregulated genes controlled by aberrant DNA methylation across multiple cancer types were primarily involved in the calcium-signaling and CAMs pathways, which are associated with oncogenesis [4, 7, 32]. Of note, genes in the calcium-signaling pathway were decreased by hypermethylation (Figure 3, Supplementary Figure 2), and were shared by multiple solid cancer types (Table 2 and Supplementary Table 7). More importantly, some epigenetically silenced genes were located in key nodes of the calcium-signaling pathway, including Na^+^/Ca^2+^ exchanger and GPCR. The calcium-signaling pathway acts in various biological processes, such as cell cycle, survival, apoptosis, migration, and gene expression [33]. Previous studies have found that the calcium-signaling pathway is deregulated in cancers, and that upregulating or downregulating the genes in the pathway can promote cancer cell proliferation, migration, and tumor metastasis [32, 34]. A recent study identified alterations in the expression of proteins involved in the movement of Ca^2+^ across the plasma membrane and subcellular organelles in cancers [35]. Changing calcium channels and pumps in cancer can trigger calcium fluxes, which are an essential component of the epigenetic mechanism of action [34]. Ca^2+^ channels or pumps are potential therapeutic targets for specific cancer subtypes and are correlated with prognosis [34–36]. In the present study, we did find that the CAMs pathway was downregulated by DNA methylation across different cancer types. This pathway is correlated with cancer angiogenesis, invasion, and metastasis, and thus cell adhesion molecules might be applied in cancer therapy [37–41]. The calcium-signaling pathway is critical in cancer biology, and as far as we know, this study is the first report to reveal the similarities in the calcium-signaling pathway and CAMs pathway in various cancer types via large-scale data mining.

DNA methylation can alter gene expression without changing gene sequences, and can be reversed by many effectors, such as the environment or drugs [5, 10, 42]. Previous studies have proposed that hypermethylation can serve as a cancer therapeutic target [5, 43]. We found more downregulated than upregulated genes across the studied cancers. Some of the epigenetically silenced genes detected in this study are involved in tumorigenesis, and could possibly be used as prognostic biomarkers [7]. DNA methylation-mediated gene silencing is essential for cancer cell survival, and demethylation of these genes by drugs can stimulate gene expression and improve patient condition [16, 44]. Since the calcium-signaling and CAMs pathways play critical roles in tumorigenesis, reversing the aberrant DNA methylation of these pathways could be an effective strategy to suppress cancers and develop anti-cancer drugs. A recent study indicated that targeting the calcium-signaling pathway can reverse epigenetic silencing of tumor suppressor genes (TSGs) in cancer by drugs [35], lending support to our notion that the calcium-signaling pathway could be an effective target in cancer therapy.

In summary, we found a common pattern of DNA methylation in gene expression regulation across multiple solid cancer types. DNA methylation generally decreased gene expression across the studied cancers, but increased expression in a few genes. This large-scale analysis revealed, for the first time, hypermethylation in the calcium-signaling pathway, which could be a promising therapeutic target in cancer treatment.

## MATERIALS AND METHODS

### Data acquisition and filtration

We collected DNA methylation data of 4,138 tumor samples and 338 matched normal tissue samples across 12 cancer types, as well as the corresponding gene expression data, from The Cancer Genome Atlas (TCGA) database (http://cancergenome.nih.gov/) (Supplementary Table 1). DNA methylation data were produced by the Illumina Infinium Human DNA Methylation 450 platform and quantified using beta values ranging from 0 to 1, with values close to 0 or close to 1 indicating low or high levels of DNA methylation, respectively. Gene expression data were produced by the Illumina HiSeq 2000 RNA Sequencing Version 2 analysis platform and quantified using RNA-Seq by Expectation-Maximization (RSEM). In each cancer type, CpG sites and genes with missing values over 5% in all samples were disregarded. The remaining CpG sites and genes with missing values in a few subjects were filled using R package “impute”. The DNA methylation β-values and gene expression RSEM values were normalized by R package “preprocessCore”. All CpG sites were annotated by R package “IlluminaHumanMethylation450k.db”.

### Genome-wide methylation analysis across 12 cancer types

We compared the genome-wide methylation levels in normal tissues to tumor tissues based on the mean DNA methylation beta value for each CpG site in each cancer type. The hypermethylated CpG sites were determined by the following criteria: CpG sites were unmethylated in normal adjacent tissue (mean DNA methylation beta value ≤ 0.2) and methylated in tumor tissue (mean DNA methylation beta value > 0.2). The hypomethylated CpG sites were determined by similar criteria: CpG sites were unmethylated in normal adjacent tissue (mean DNA methylation beta value > 0.2) and methylated in tumor tissue (mean DNA methylation beta value ≤ 0.2) [45]. To explore the distribution of these hypermethylated and hypomethylated CpG sites in the genome, we annotated the CpG sites using the “IlluminaHumanMethylation450k.db” package.

### Association of DNA methylation with gene expression across 12 cancer types

We examined the relationship between DNA methylation and gene expression across cancers using Pearson correlation. A Pearson correlation coefficient value of less than -0.2 and *p*-value less than 0.05 were considered statistically significant [45]. To identify the candidate epigenetically silenced genes, we adopted the following criteria: 1) 95^th^ percentile for DNA methylation beta value in normal tissue < 0.2; 2) 95^th^ percentile for DNA methylation beta value in tumor tissue > 0.2 and maximum DNA methylation beta value in tumor tissue > 0.5; and 3) mean DNA methylation beta value in tumor tissue > 0.2. To identify the candidate epigenetically activated genes, we adopted the following criteria: 1) 95^th^ percentile for DNA methylation beta value in tumor tissue < 0.2; 2) 95^th^ percentile for DNA methylation beta value in normal tissue > 0.2 and maximum DNA methylation beta value in normal tissue > 0.5; and 3) mean DNA methylation beta value in normal tissue > 0.2.

### Protein interaction and pathway enrichment analysis

Protein-protein interactions were analyzed using the STRING database (http://string-db.org/). To understand the pathways involved in the dysregulated genes across cancers, we explored the biological pathways using the Kyoto Encyclopedia of Genes and Genomes (KEGG) pathway database (http://www.kegg.jp/kegg/pathway.html), which is widely used for systematic analysis of gene function [46]. All pathways were downloaded from the KEGG pathway database [47], and cluster analysis using the Database for Annotation, Visualization and Integrated Discovery (DAVID) (https://david.ncifcrf.gov/) was conducted [48]. A *p*-value of less than 0.05 was considered statistically significant.

## SUPPLEMENTARY MATERIALS FIGURES AND TABLES




