# Supplementary material for: Large-scale DNA methylation expression analysis across 12 solid cancers reveals hypermethylation in the calcium-signaling pathway
Source: Oncotarget. 2017 Jan 2;8(7):11868–76. doi: 10.18632/oncotarget.14417 (PMC5355310; doi:10.18632/oncotarget.14417)
Supplement: Supplementary file 1 [file oncotarget-08-11868-s001.pdf]

Large-scale DNA methylation expression analysis across 12 solid cancers reveals hypermethylation in the calcium-signaling pathway

SUPPLEMENTARY FIGURES AND TABLES

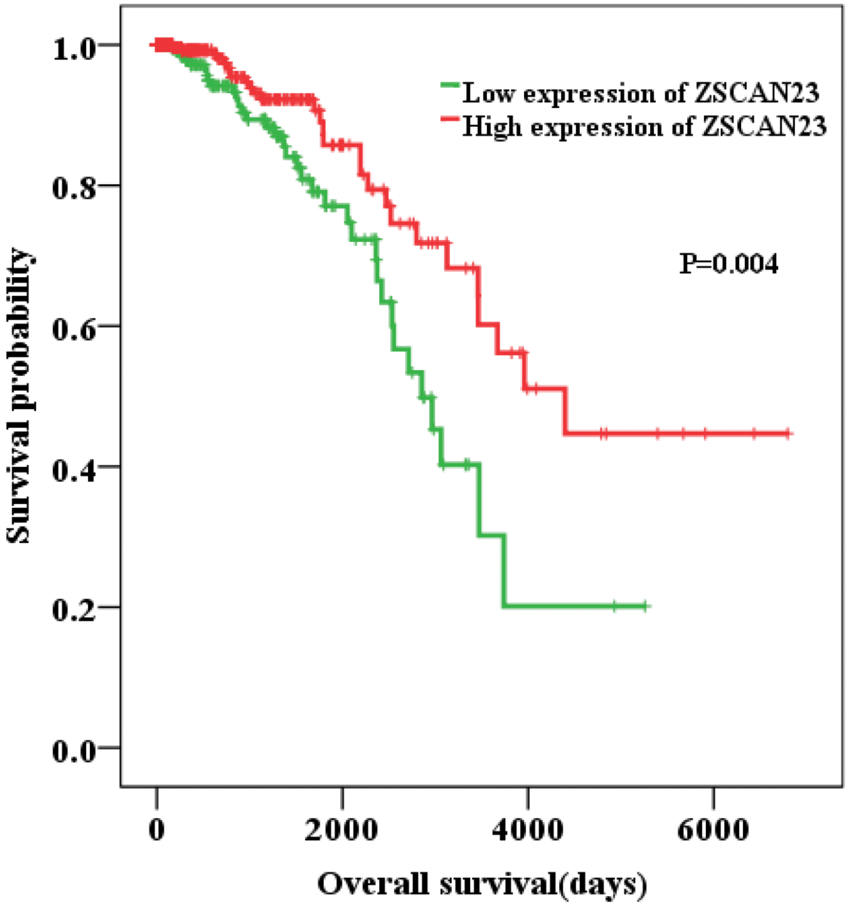

Supplementary Figure 1: Association of *ZSCAN23* gene expression with overall survival in breast cancer.

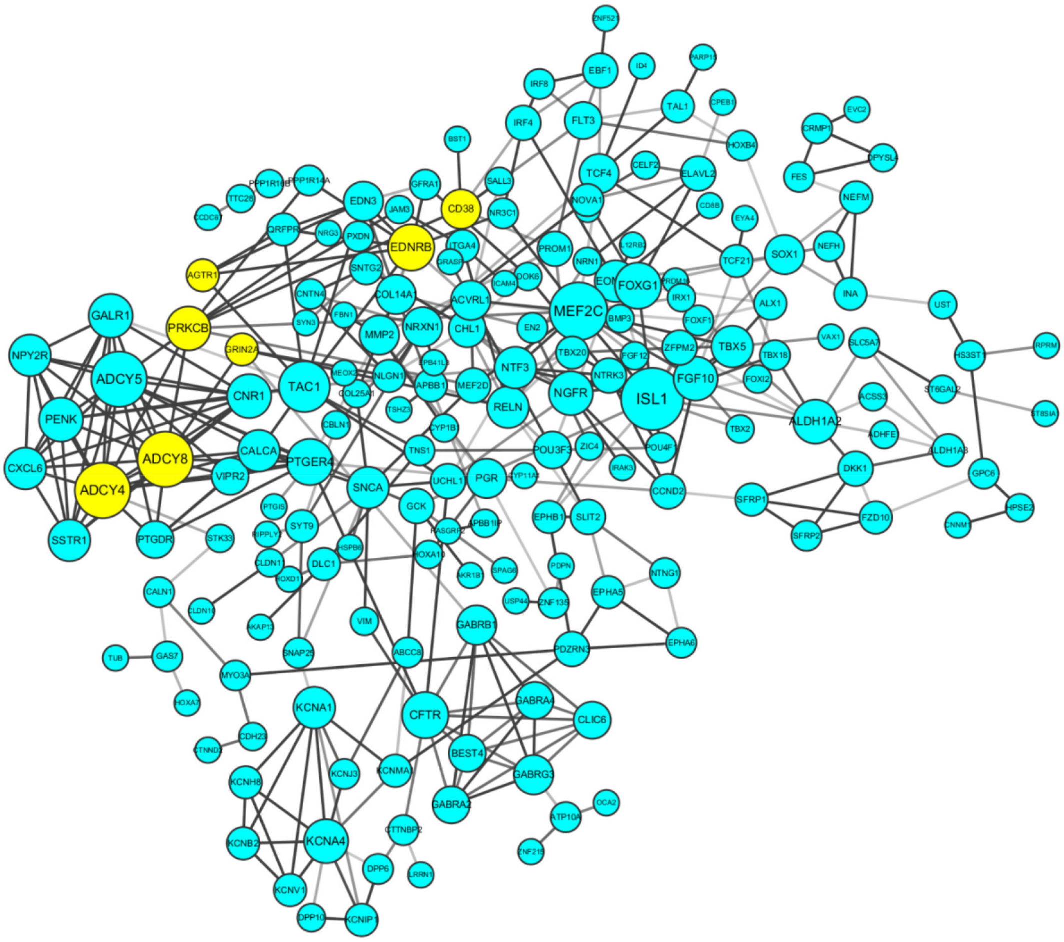

Supplementary Figure 2: Interactional network based on 340 epigenetically silenced genes in at least five cancer types. Genes in yellow circles, including *ADCY4*, *ADCY8*, *PRKCB*, *AGTR1*, *EDNRB*, *CD38* and *GRIN2A*, are involved in the calcium-signaling pathway and are located in the center of the network. Color of lines represents interaction strength between proteins.

Supplementary Table 1: Data on samples downloaded from TCGA

Cancer type	Cancer name	Tumor sample	Normal sample
BLCA	Bladder urothelial carcinoma	249	17
BRCA	Breast invasive carcinoma	711	68
COAD	Colon adenocarcinoma	251	19
HNSC	Head and neck squamous cell carcinoma	494	20
KIRC	Kidney renal clear cell carcinoma	300	24
KIRP	Kidney renal papillary cell carcinoma	181	23
LIHC	Liver hepatocellular carcinoma	197	41
LUAD	Lung adenocarcinoma	427	21
LUSC	Lung squamous cell carcinoma	353	8
PRAD	Prostate adenocarcinoma	327	34
THCA	Thyroid carcinoma	493	50
UCEC	Uterine corpus endometrial carcinoma	155	13
Total		4138	338

Supplementary Table 2: Ratio between hyper- and hypomethylated annotated CpG sites in gene-related regions across cancers

Cancer types	1st Exon	3'UTR	5'UTR	Gene body	TSS200	TSS1500
BLCA	7.84931507	3.290909091	3.12946429	3.007945516	3.197002141	2.25624179
BRCA	7.75	2.846153846	3.59322034	2.808429119	3.442176871	2.18577075
COAD	7.2625	1.689189189	3.3697479	2.588691796	3.254826255	2.35595391
HNSC	7.67692308	1.689655172	2.87559809	2.050884956	2.961702128	1.71668823
KIRC	6.68085106	0.774193548	1.62916667	1.486044273	2.31092437	1.53376206
KIRP	4.89795918	3.238095238	2.08426966	2.693163752	2.283636364	2.36868687
LIHC	11.2884615	4.555555556	4.83064516	4.570075758	7.755760369	4.37288136
LUAD	6.47826087	1.967741935	2.41015625	2.04048583	3.008474576	1.5237581
LUSC	4.56521739	2.274193548	2.16666667	2.035246727	2.077262693	1.2553916
PRAD	3.51685393	0.849462366	2.02290076	1.335083115	2.257471264	1.21586931
THCA	9.2	3.75	1.91346154	3.074183976	4.092783505	3.45789474
UCEC	10.2040816	3.396551724	3.26548673	2.928057554	4.247252747	2.05988024

Supplementary Table 3: Examples of negative correlation between DNA methylation and gene expression across cancers

See Supplementary File 1

Supplementary Table 4: Number of epigenetically silenced genes across 12 cancer types

Cancer types	No. of genes
9	6
8	37
7	96
6	190
5	340
4	606
3	996
2	1664
1	3079

Supplementary Table 5: Six epigenetically silenced genes across nine cancer types

Genes	Cancer types
NOVA1	BLCA, BRCA, COAD, HNSC, LIHC, LUAD, LUSC, PRAD, UCEC
NRXN1	BLCA, BRCA, HNSC, KIRC, KIRP, LUAD, LUSC, PRAD, UCEC
TMEM132C	BLCA, BRCA, COAD, HNSC, LIHC, LUAD, LUSC, PRAD, UCEC
USP44	BLCA, BRCA, COAD, HNSC, KIRC, KIRP, LIHC, LUSC, UCEC
VIPR2	BLCA, BRCA, COAD, HNSC, KIRC, LIHC, LUAD, LUSC, UCEC
ZSCAN23	BLCA, BRCA, COAD, HNSC, KIRC, LIHC, LUAD, LUSC, UCEC

Supplementary Table 6: Four genes epigenetically activated by DNA methylation across two cancer types

Genes	Cancer types
GRHL2	BRCA, UCEC
NAA25,	KIRC, KRP
NOD2	KIRC, KRP
TNFRSF9	KIRC, KRP

Supplementary Table 7: Pathway enrichment based on 340 epigenetically silenced genes in at least five cancer types

Pathways	P-value
hsa04080: Neuroactive ligand-receptor interaction	4.23E-04
hsa04020: Calcium-signaling pathway	0.002226747
hsa04514: Cell adhesion molecules (CAMs) pathway	0.004179564
hsa04270: Vascular smooth muscle contraction	0.022289836
hsa04916: Melanogenesis	0.044549793

Supplementary Table 8: Eight genes epigenetically silenced in the calcium-signaling pathway in at least five cancer types

Genes	Cancer types
AGTR1	BLCA, BRCA, COAD, HNSC, LUAD, UCEC
GRIN2A	BLCA, BRCA, COAD, HNSC, LUAD, UCEC
ITPKB	COAD, KIRP, LIHC, LUAD, LUSC, UCEC
SLC8A3	BLCA, COAD, KIRP, LUAD, LUSC, UCEC
ADCY4	BRCA, COAD, LUAD, LUSC, UCEC
ADCY8	BRCA, HNSC, LUAD, LUSC, UCEC
BST1	BLCA, BRCA, LUAD, LUSC, UCEC
PRKCB	BRCA, COAD, LIHC, LUAD, UCEC

Supplementary Table 9: Gene expression fold change (tumor/normal tissue) in the calcium-signaling pathway across 12 cancer types

Cancer types	*ADCY4*	*ADCY8*	*AGTR1*	*BST1*	*GRIN2A*	*ITPKB*	*PRKCB*	*SLC8A3*
BLCA	0.42	3.27	0.11	0.74	0.53	0.26	0.22	0.14
BRCA	0.35	1.35	1.20	0.44	0.79	0.79	1.43	0.56
COAD	1.06	0.53	0.18	0.36	0.22	0.63	0.26	0.32
HNSC	0.74	0.15	0.38	0.86	2.72	0.98	1.34	0.27
KIRC	1.80	58.89	1.30	1.55	3.91	0.50	0.89	3.39
KIRP	0.25	1.41	0.14	0.78	0.42	0.32	1.59	3.18
LIHC	1.74	12.35	0.39	0.76	9.58	1.07	0.47	2.02
LUAD	0.25	0.08	0.27	0.39	2.67	0.73	0.60	0.36
LUSC	0.15	2.29	0.13	0.39	6.11	0.79	0.38	0.18
PRAD	1.21	0.03	2.84	0.77	0.42	0.87	0.29	1.15
THCA	1.19	112.82	0.48	0.75	1.49	1.40	0.80	1.07
UCEC	0.22	0.03	0.26	0.34	0.09	0.31	0.26	0.99
