# Supplementary material for: Large-scale DNA methylation expression analysis across 12 solid cancers reveals hypermethylation in the calcium-signaling pathway
Source: Oncotarget. 2017 Jan 2;8(7):11868–76. doi: 10.18632/oncotarget.14417 (PMC5355310; doi:10.18632/oncotarget.14417)
Supplement: Supplementary file 2 [file oncotarget-08-11868-s002.doc]

**Table S3. Examples of negative correlation between DNA methylation and gene expression across cancers**

| CpG sites | Correlation coefficient value | P-value | Genes | Cancer types |
| --- | --- | --- | --- | --- |
| cg00651523 | -0.441182631 | 3.07E-14 | *ZSCAN23* | BLCA |
| cg06818710 | -0.47021605 | 4.19E-44 | *ZSCAN23* | BRCA |
| cg07836142 | -0.396349417 | 1.05E-30 | *ZSCAN23* | BRCA |
| cg07838603 | -0.438326353 | 6.53E-38 | *ZSCAN23* | BRCA |
| cg09682330 | -0.563383549 | 1.90E-66 | *ZSCAN23* | BRCA |
| cg13525197 | -0.476618538 | 1.99E-45 | *ZSCAN23* | BRCA |
| cg21486944 | -0.376020551 | 1.07E-10 | *ZSCAN23* | COAD |
| cg24703168 | -0.387457032 | 2.56E-11 | *ZSCAN23* | COAD |
| cg07836142 | -0.585972078 | 1.03E-48 | *ZSCAN23* | HNSC |
| cg07838603 | -0.56132394 | 5.24E-44 | *ZSCAN23* | HNSC |
| cg09682330 | -0.603068551 | 3.19E-52 | *ZSCAN23* | HNSC |
| cg14547644 | -0.48561155 | 1.08E-20 | *ZSCAN23* | KIRC |
| cg06818710 | -0.332603516 | 1.48E-07 | *ZSCAN23* | LIHC |
| cg13525197 | -0.281658853 | 1.02E-05 | *ZSCAN23* | LIHC |
| cg24703168 | -0.307724972 | 1.30E-06 | *ZSCAN23* | LIHC |
| cg00651523 | -0.589281408 | 3.11E-43 | *ZSCAN23* | LUAD |
| cg06818710 | -0.572367909 | 2.39E-40 | *ZSCAN23* | LUAD |
| cg09682330 | -0.633769056 | 1.08E-51 | *ZSCAN23* | LUAD |
| cg13525197 | -0.580715056 | 9.44E-42 | *ZSCAN23* | LUAD |
| cg00651523 | -0.558256685 | 5.77E-31 | *ZSCAN23* | LUSC |
| cg13525197 | -0.522459091 | 1.13E-26 | *ZSCAN23* | LUSC |
| cg14547644 | -0.526899897 | 3.53E-27 | *ZSCAN23* | LUSC |
| cg06818710 | -0.672970874 | 1.66E-23 | *ZSCAN23* | UCEC |
| cg07836142 | -0.652837518 | 8.92E-22 | *ZSCAN23* | UCEC |
| cg07838603 | -0.65469772 | 6.25E-22 | *ZSCAN23* | UCEC |
| cg09682330 | -0.68374274 | 1.73E-24 | *ZSCAN23* | UCEC |
| cg13525197 | -0.645394762 | 3.61E-21 | *ZSCAN23* | UCEC |
| cg21486944 | -0.624246982 | 1.56E-19 | *ZSCAN23* | UCEC |
| cg24703168 | -0.625128985 | 1.34E-19 | *ZSCAN23* | UCEC |
| cg03976877 | -0.301198522 | 4.79E-07 | *VIPR2* | BLCA |
| cg18349835 | -0.302070681 | 4.42E-07 | *VIPR2* | BLCA |
| cg19814400 | -0.229270373 | 0.000148565 | *VIPR2* | BLCA |
| cg21038156 | -0.307925089 | 2.57E-07 | *VIPR2* | BLCA |
| cg19814400 | -0.247283547 | 2.56E-12 | *VIPR2* | BRCA |
| cg20673829 | -0.489756822 | 3.10E-48 | *VIPR2* | BRCA |
| cg21038156 | -0.302670099 | 5.74E-18 | *VIPR2* | BRCA |
| cg03976877 | -0.304955573 | 2.38E-07 | *VIPR2* | COAD |
| cg18349835 | -0.240660238 | 5.36E-05 | *VIPR2* | COAD |
| cg19814400 | -0.246378592 | 3.50E-05 | *VIPR2* | COAD |
| cg03976877 | -0.242312588 | 2.65E-08 | *VIPR2* | HNSC |
| cg18349835 | -0.319746125 | 1.11E-13 | *VIPR2* | HNSC |
| cg19814400 | -0.214469726 | 9.21E-07 | *VIPR2* | HNSC |
| cg20673829 | -0.250204858 | 8.90E-09 | *VIPR2* | HNSC |
| cg21038156 | -0.282128402 | 7.34E-11 | *VIPR2* | HNSC |
| cg23572908 | -0.241298052 | 1.06E-05 | *VIPR2* | KIRC |
| cg03976877 | -0.348290891 | 3.42E-08 | *VIPR2* | LIHC |
| cg04525189 | -0.319117558 | 4.92E-07 | *VIPR2* | LIHC |
| cg13794530 | -0.461991218 | 5.50E-14 | *VIPR2* | LIHC |
| cg18349835 | -0.378914213 | 1.53E-09 | *VIPR2* | LIHC |
| cg20673829 | -0.349208869 | 3.13E-08 | *VIPR2* | LIHC |
| cg20830514 | -0.315059126 | 6.98E-07 | *VIPR2* | LIHC |
| cg21038156 | -0.36635975 | 5.68E-09 | *VIPR2* | LIHC |
| cg25189564 | -0.373315725 | 2.76E-09 | *VIPR2* | LIHC |
| cg03976877 | -0.2866482 | 6.39E-10 | *VIPR2* | LUAD |
| cg13794530 | -0.317850127 | 5.65E-12 | *VIPR2* | LUAD |
| cg20673829 | -0.308530456 | 2.46E-11 | *VIPR2* | LUAD |
| cg25189564 | -0.306626933 | 3.30E-11 | *VIPR2* | LUAD |
| cg21038156 | -0.217177966 | 3.16E-05 | *VIPR2* | LUSC |
| cg03976877 | -0.201919098 | 0.008671953 | *VIPR2* | UCEC |
| cg13794530 | -0.21306002 | 0.005556488 | *VIPR2* | UCEC |
| cg18349835 | -0.277703148 | 0.00026789 | *VIPR2* | UCEC |
| cg19814400 | -0.32835581 | 1.39E-05 | *VIPR2* | UCEC |
| cg21038156 | -0.385571935 | 2.45E-07 | *VIPR2* | UCEC |
| cg03308628 | -0.410666094 | 2.28E-12 | *USP44* | BLCA |
| cg07783282 | -0.392253586 | 2.51E-11 | *USP44* | BLCA |
| cg22538054 | -0.442695054 | 2.45E-14 | *USP44* | BLCA |
| cg23982858 | -0.430513651 | 1.45E-13 | *USP44* | BLCA |
| cg03308628 | -0.41076439 | 4.58E-33 | *USP44* | BRCA |
| cg13879483 | -0.5795574 | 4.30E-71 | *USP44* | BRCA |
| cg17368254 | -0.415250948 | 7.98E-34 | *USP44* | BRCA |
| cg22538054 | -0.426699042 | 8.20E-36 | *USP44* | BRCA |
| cg07783282 | -0.26226223 | 1.01E-05 | *USP44* | COAD |
| cg22538054 | -0.210909334 | 0.000419106 | *USP44* | COAD |
| cg23982858 | -0.238127178 | 6.45E-05 | *USP44* | COAD |
| cg03308628 | -0.509249913 | 2.89E-35 | *USP44* | HNSC |
| cg22538054 | -0.528608371 | 2.44E-38 | *USP44* | HNSC |
| cg23982858 | -0.493214154 | 7.27E-33 | *USP44* | HNSC |
| cg07783282 | -0.343270414 | 1.91E-10 | *USP44* | KIRC |
| cg13879483 | -0.420232592 | 2.22E-15 | *USP44* | KIRC |
| cg22802813 | -0.459331437 | 2.03E-18 | *USP44* | KIRC |
| cg23982858 | -0.462544965 | 1.10E-18 | *USP44* | KIRC |
| cg07783282 | -0.20394627 | 0.003435157 | *USP44* | KIRP |
| cg13879483 | -0.258716488 | 0.000186734 | *USP44* | KIRP |
| cg03308628 | -0.412380265 | 3.46E-11 | *USP44* | LIHC |
| cg07783282 | -0.473098461 | 1.12E-14 | *USP44* | LIHC |
| cg17368254 | -0.294046575 | 3.93E-06 | *USP44* | LIHC |
| cg22538054 | -0.44793952 | 3.80E-13 | *USP44* | LIHC |
| cg23982858 | -0.453207328 | 1.86E-13 | *USP44* | LIHC |
| cg13879483 | -0.560429217 | 5.04E-16 | *USP44* | LUAD |
| cg07783282 | -0.478190791 | 5.02E-22 | *USP44* | LUSC |
| cg13879483 | -0.524704841 | 6.28E-27 | *USP44* | LUSC |
| cg13879483 | -0.320046125 | 1.09E-13 | *USP44* | PRAD |
| cg13879483 | -0.280128402 | 7.14E-11 | *USP44* | THCA |
| cg17368254 | -0.560429007 | 2.80E-15 | *USP44* | UCEC |
| cg22802813 | -0.572609062 | 5.08E-16 | *USP44* | UCEC |
| cg00765828 | -0.267913859 | 8.39E-09 | *TMEM132D* | LUAD |
| cg04733537 | -0.450498618 | 8.93E-24 | *TMEM132D* | LUAD |
| cg23891360 | -0.369230676 | 6.44E-16 | *TMEM132D* | LUAD |
| cg06164660 | -0.282785313 | 2.44E-06 | *TMEM132C* | BLCA |
| cg13824555 | -0.267728361 | 8.50E-06 | *TMEM132C* | BLCA |
| cg26682580 | -0.292457402 | 1.05E-06 | *TMEM132C* | BLCA |
| cg03530754 | -0.360810367 | 2.31E-25 | *TMEM132C* | BRCA |
| cg04475027 | -0.398494584 | 4.75E-31 | *TMEM132C* | BRCA |
| cg06164660 | -0.375729661 | 1.59E-27 | *TMEM132C* | BRCA |
| cg10541517 | -0.337412007 | 3.40E-22 | *TMEM132C* | BRCA |
| cg11877129 | -0.340351993 | 1.41E-22 | *TMEM132C* | BRCA |
| cg13824555 | -0.283289308 | 7.65E-16 | *TMEM132C* | BRCA |
| cg26682580 | -0.378152494 | 6.90E-28 | *TMEM132C* | BRCA |
| cg06164660 | -0.26751516 | 7.14E-10 | *TMEM132C* | HNSC |
| cg10541517 | -0.311806156 | 4.72E-13 | *TMEM132C* | HNSC |
| cg13824555 | -0.25862824 | 2.67E-09 | *TMEM132C* | HNSC |
| cg26682580 | -0.287357293 | 3.15E-11 | *TMEM132C* | HNSC |
| cg04475027 | -0.24371736 | 0.000146155 | *TMEM132C* | LIHC |
| cg10406124 | -0.247831527 | 0.000111693 | *TMEM132C* | LIHC |
| cg03530754 | -0.225662899 | 1.40E-06 | *TMEM132C* | LUAD |
| cg04475027 | -0.285526256 | 7.50E-10 | *TMEM132C* | LUAD |
| cg06164660 | -0.269849638 | 6.49E-09 | *TMEM132C* | LUAD |
| cg10541517 | -0.247672742 | 1.09E-07 | *TMEM132C* | LUAD |
| cg11877129 | -0.201699628 | 1.70E-05 | *TMEM132C* | LUAD |
| cg13824555 | -0.254426602 | 4.75E-08 | *TMEM132C* | LUAD |
| cg26682580 | -0.27834679 | 2.05E-09 | *TMEM132C* | LUAD |
| cg03530754 | -0.220434837 | 2.38E-05 | *TMEM132C* | LUSC |
| cg04475027 | -0.260804027 | 5.03E-07 | *TMEM132C* | LUSC |
| cg10541517 | -0.21259901 | 4.66E-05 | *TMEM132C* | LUSC |
| cg11877129 | -0.20959711 | 5.99E-05 | *TMEM132C* | LUSC |
| cg26682580 | -0.249460106 | 1.59E-06 | *TMEM132C* | LUSC |
| cg10541517 | -0.206845432 | 7.69E-05 | *TMEM132C* | PRAD |
| cg26682580 | -0.423026246 | 4.62E-17 | *TMEM132C* | PRAD |
| cg00579520 | -0.566601948 | 1.19E-15 | *TMEM132C* | UCEC |
| cg02543772 | -0.346337801 | 4.25E-06 | *TMEM132C* | UCEC |
| cg03530754 | -0.449691671 | 9.66E-10 | *TMEM132C* | UCEC |
| cg04475027 | -0.415909034 | 2.06E-08 | *TMEM132C* | UCEC |
| cg06164660 | -0.396067502 | 1.07E-07 | *TMEM132C* | UCEC |
| cg08774452 | -0.297601367 | 8.94E-05 | *TMEM132C* | UCEC |
| cg10406124 | -0.417474027 | 1.80E-08 | *TMEM132C* | UCEC |
| cg10541517 | -0.423922114 | 1.03E-08 | *TMEM132C* | UCEC |
| cg11265160 | -0.250931819 | 0.001035062 | *TMEM132C* | UCEC |
| cg11877129 | -0.398238433 | 8.99E-08 | *TMEM132C* | UCEC |
| cg13824555 | -0.375414604 | 5.32E-07 | *TMEM132C* | UCEC |
| cg18628094 | -0.368360101 | 8.98E-07 | *TMEM132C* | UCEC |
| cg26682580 | -0.410754052 | 3.20E-08 | *TMEM132C* | UCEC |
| cg17677030 | -0.244682314 | 4.99E-05 | *NRXN1* | BLCA |
| cg25717438 | -0.259854259 | 1.59E-05 | *NRXN1* | BLCA |
| cg27112565 | -0.221378797 | 0.000252725 | *NRXN1* | BLCA |
| cg25717438 | -0.360631964 | 2.45E-25 | *NRXN1* | BRCA |
| cg27112565 | -0.296415518 | 2.90E-17 | *NRXN1* | BRCA |
| cg17526573 | -0.208267872 | 1.91E-06 | *NRXN1* | HNSC |
| cg13906811 | -0.210617594 | 0.002496813 | *NRXN1* | KIRP |
| cg17526573 | -0.211105205 | 6.59E-06 | *NRXN1* | LUAD |
| cg17526573 | -0.217810282 | 2.99E-05 | *NRXN1* | LUSC |
| cg03295083 | -0.205522134 | 8.57E-05 | *NRXN1* | PRAD |
| cg25717438 | -0.231515002 | 0.002531872 | *NRXN1* | UCEC |
| cg18488855 | -0.383121842 | 7.81E-11 | *NOVA1* | BLCA |
| cg20478129 | -0.338929951 | 1.18E-08 | *NOVA1* | BLCA |
| cg19832521 | -0.530531398 | 9.14E-58 | *NOVA1* | BRCA |
| cg07559273 | -0.471443118 | 1.12E-16 | *NOVA1* | COAD |
| cg15602241 | -0.440828841 | 1.50E-14 | *NOVA1* | COAD |
| cg16791424 | -0.548109191 | 4.82E-23 | *NOVA1* | COAD |
| cg18468511 | -0.418828459 | 3.78E-13 | *NOVA1* | COAD |
| cg18488855 | -0.499646478 | 7.78E-19 | *NOVA1* | COAD |
| cg20478129 | -0.41641012 | 5.32E-13 | *NOVA1* | COAD |
| cg20478129 | -0.358468821 | 4.96E-17 | *NOVA1* | HNSC |
| cg18488855 | -0.298166933 | 2.83E-06 | *NOVA1* | LIHC |
| cg19832521 | -0.358385194 | 1.27E-08 | *NOVA1* | LIHC |
| cg19832521 | -0.364850404 | 1.49E-15 | *NOVA1* | LUAD |
| cg19832521 | -0.331066407 | 1.11E-10 | *NOVA1* | LUSC |
| cg02014003 | -0.503924518 | 3.33E-12 | *NOVA1* | UCEC |
| cg07559273 | -0.546476723 | 1.82E-14 | *NOVA1* | UCEC |
| cg15602241 | -0.482133057 | 3.66E-11 | *NOVA1* | UCEC |
| cg18488855 | -0.612254533 | 1.17E-18 | *NOVA1* | UCEC |
| cg19832521 | -0.661040626 | 1.83E-22 | *NOVA1* | UCEC |
| cg20478129 | -0.497050878 | 7.22E-12 | *NOVA1* | UCEC |
| cg20961943 | -0.38243361 | 3.13E-07 | *NOVA1* | UCEC |
